# Factors associated with intrapartum stillbirth in a tertiary teaching hospital in Burkina Faso

**DOI:** 10.3389/fgwh.2023.1038817

**Published:** 2023-04-03

**Authors:** Bewendin Evelyne Komboigo, Hyacinthe Zamane, Abou Coulibaly, Sansan Rodrigue Sib, Madina Thiombiano, Blandine Thieba

**Affiliations:** ^1^ Higher Institute of Health Sciences, Nazi Boni University, Bobo-Dioulasso, Burkina Faso; ^2^ Department of Gynecology, Obstetrics and Reproductive Medicine, Sourô Sanou University Hospital Center, Bobo-Dioulasso, Burkina Faso; ^3^ Health Science Training and Research Unit, Joseph Ki-Zerbo University, Ouagadougou, Burkina Faso; ^4^ Department of Gynecology-Obstetrics, Yalgado Ouedraogo Teaching Hospital, Ouagadougou, Burkina Faso; ^5^ Research Institute of Health Sciences (IRSS), Ouagadougou, Burkina Faso; ^6^ Obstetrics Gynecology Department, Regional Teaching Hospital of Ouahygouya, Ouahigouya, Burkina Faso; ^7^ Department of Gynecology-Obstetrics, Regional Hospital Center of Koudougou, Koudougou, Burkina Faso

**Keywords:** stillbirths, risk factors, intrapartum, hospital, Burkina Faso

## Abstract

**Introduction:**

Intrapartum stillbirth is an indicator of health and community development.

**Objective:**

To identify the risk factors associated with intrapartum stillbirth in a tertiary teaching hospital in Burkina Faso.

**Patients and methods:**

A case-control study conducted from January 1 to August 30, 2019. Cases were defined as patients admitted to Yalgado Ouedraogo teaching hospital (YOTH) with a live fetus of at least 28 weeks’ gestation and who gave birth to an intrapartum stillborn, a fetus delivered without any signs of life in the first minute postpartum. Controls were defined as patients who delivered a live newborn. Study controls were gradually recruited and matched to cases. For each case, two controls were recruited and matched according to criteria such as delivery route and day of delivery. Data were cleaned in Epidata and exported to Stata for analysis. Variables with a *p* < 0.05 significance level in the multivariable regression were retained. Odds ratio (OR) and 95% confidence intervals are reported.

**Results:**

Eighty-three intrapartum stillbirths were documented among a total of 4,122 deliveries, a stillbirth rate of 20.1 per 1,000 births. There was a statistically significant association between intrapartum stillbirth and prior caesarean section (*p* = 0.045), multiparity (*p* = 0.03), the receipt of antenatal care (ANC) by a nurse (*p* = 0.005) and the disuse of the partogram (*p* = 0.004). We did not find a significant association between the number of ANC consultations performed (*p* = 0.3), whether membranes were ruptured at admission (*p* = 0.6), the duration of labor (*p* = 0.6) and intrapartum fetal death. Multivariate analysis showed that patient referral to another heath facility (OR: 3.33; 95% IC: 1.56, 7.10), no obstetric ultrasound performed (OR: 3.16; 95% IC: 2.11, 4.73), birth weight less than 2,500 g (OR: 7.49; 95% IC: 6.40, 8.76) were significantly associated with intrapartum stillbirth.

**Conclusion:**

Specific interventions must be taken to identify these risk factors of intrapartum stillbirth in order to ensure better and appropriate management.

## 1. Introduction

Stillbirths are an indicator of the health and development of a community (1). In the worldwide, the intrapartum mortality rate varies from region to region. Stillbirth has decreased significantly in developed countries due to the per partum component, likely a result of better access to care (2). This rate seems higher when the socio-economic level of the community is low. In 2006, according to the World Health Organization (WHO), in Africa 7.5% of fetuses die during the perinatal period, including 4.1% in the intrapartum period and 3.4% in the early neonatal period. This is a reflection of the low level of health (3). Significant progress remains to be made because the figures are still high in Sub-Saharan Africa (4, 5). These rates are the indicators that best reflect the quality of prenatal consultations and labor monitoring (6). There is very little data on stillbirths in Burkina Faso, like many countries in sub-Saharan Africa, but has high rates of intrapartum deaths (7–10) and the intrapartum stillbirth rate is disparate from one region to another. In 2018, according to data from the Department of Health and Family, the per partum death rate was very high in the Centre (41.4%), Centre-Ouest (48.1%) and Haut-Bassins regions (43.7%) (11). It was the ratio of fresh stillbirths to the total number of stillbirths in these regions. In the obstetrics department of Yalgado Ouedraogo teaching hospital in Ouagadougou (YOTH), the intrapartum stillbirth rate remains high (63.4% of all stillbirths) (11). It was the ratio of fresh stillbirths to the total. In order to prevent future death, the present study aims to identify the factors associated with intrapartum stillbirth at YOTH.

## 2. Patients and method

The study took place in the Obstetrics department of the Yalgado Ouedraogo University Hospital Center in Ouagadougou (YOTH), Burkina Faso. A prospective case-control study was conducted of intrapartum stillbirth over a period of 8 months from January 1 to August 30, 2019. Women in labor carrying an active pregnancy of at least 28 weeks’ gestation were eligible for the study. All patients who delivered an intrapartum stillborn during the study period were included. Cases were defined as patients admitted to CHU-YO with a live fetus of at least 28 weeks’ gestation and who gave birth to an intrapartum stillborn, a fetus delivered without any signs of life in the first minute postpartum. Controls were defined as patients who delivered a live newborn. Study controls were gradually recruited and matched to cases. For each case, two controls were recruited and matched according to criteria such as delivery route and day of delivery. Antepartum stillbirths as well as live births with present cardiac activity, yet who died afterwards were excluded from the study. In addition, patients who delivered at home or in route to the health facility were not included. Pre-existing health conditions of women during pregnancy were taken into account in the exclusion criteria.

Interviews with patients and register and record review were conducted using individual data collection forms. Data collection was carried out by students specializing in the said department. The mothers of stillbirths were informed that this was a study and their consent was taken before the administration of the questionnaire. This was a prospective collection and informed consent was obtained before the administration of the questionnaire. The anonymity of the respondents was respected. The study had the approval of the ethics committee of Burkina Faso by deliberation number 2018-03-54. Data were collected in the postpartum.

Data was entered using Epi Data software and imported into Stata software for analysis. Logistic regression was conducted to assess bivariate associations between stillbirth and risk factors. Multivariable logistic regression using a bottom-up step-by-step method was employed to assess the relationship between stillbirth and risk factors; all variables with a *p*-value less than 0.2 during the univariate analysis were gradually included in the multivariate model. Variables with a *p* < 0.05 significance level in the multivariable regression were retained. The Hosmer and Lemeshow test was conducted to assess goodness of fit. The value of the area under the Receiver Operating Characteristic (ROC) curve was also used to assess the model's level of prediction. Taking into account the matching criteria of day of delivery, analyses corrected for a possible cluster effect. Odds ratio (OR) and 95% confidence intervals are reported.

## 3. Results

During the study period, eligibility was assessed among 4,122 deliveries. In total, data was collected on 83 cases of intrapartum stillbirth and 163 controls. Among a total of 4,122 deliveries, the reported intrapartum stillbirth rate was 20.1 per 1,000 births.

The mean age of the patients was 26.9 years. Patients under the age of 20 accounted for 19.3% (16) in the case group vs. 14% (23) in the control group.

Table 1 presents the sociodemographic factors associated with intrapartum stillbirth and Table 2 with logistic regression.

**Table 1:** Sociodemographic factors associated with intrapartum stillbirth.

Sociodemographic variables	Cases (*n* = 83)		Controls (*n* = 166)	
	*n*	%	*n*	%
Place of residence				
Rural	24	28.9	35	20.7
Urban	59	71.1	131	79.3
Marital status				
No married	57	68.7	41	24.4
Married	26	31.3	125	75.6
Occupation				
Homemaker	67	80.7	100	60.4
Civil servant/Shopkeeper/Student	16	19.3	66	39.6
Education level				
No formal education	41	49.4	45	26.8
Some formal education	42	50.6	121	73.2
Means of transportation to reach the hospital				
Ambulance/Emergency transportation	49	59	71	42.7
Personal or private means	34	41	95	57.3

**Table 2:** Sociodemographic factors associated with intrapartum stillbirth with logistic regression.

Sociodemographic variables	OR (95% IC)	*p* value
Place of residence		
Rural		
Urban	0.64 (0.35; 1.17)	0.153
Marital status		
Not married		
Married	0.70 (0.39; 1.26)	0.246
Occupation		
Homemaker		
Civil servant/Shopkeeper/Student	0.36 (0.19; 0.68)	0.002
Education level		
No formal education		
Some formal education	0.37 (0.21; 0.65)	0.001
Means of transportation to reach the hospital		
Ambulance/Emergency transportation		
Personal or private means	0.51 (0.30; 0.88)	0.016

There was a statistically significant association between intrapartum stillbirth and prior caesarean section (*p* = 0.045) and multiparity (*p* = 0.03). However, there was not a statistically significant association between the use of contraceptives and intrapartum stillbirth (*p* = 0.6). There was a statistically significant association between intrapartum fetal death and the receipt of antenatal care (ANC) by a nurse (*p* = 0.005) and the disuse of the partogram (*p* = 0.004). We did not find a significant association between the number of ANC consultations performed (*p* = 0.3), whether membranes were ruptured at admission (*p* = 0.6), the duration of labor (*p* = 0.6) and intrapartum fetal death.

There was a statistically significant association between fetal weight of less than 2,500 grams and intrapartum fetal death (*p* < 0.001). However, there was no statistically significant association between fetal presentation or position, other than the vertex presentation, and intrapartum fetal death (*p* = 0.47).

There was a statistical association between the person who decided on the consultation (in-laws/ancestors, spouse) and stillbirths (*p* < 0.001). Table 3 presents the association between the delay in making the decision to consult and intrapartum stillbirth.

### 3.1. Delay between decision to refer and arrival at YOTH

**Table 3:** Association between decision-making time to consult and intrapartum stillbirth.

Time between the decision to seek care and arrival at the heath center CSPS	OR	95% IC		*p*-value
Came directly to hospital				
Less than an hour	5.05	1.86	13.75	0.002
1–2 h	3.56	1.40	9.04	0.008
>2 h	6.85	2.76	17.03	<0.001

There was a statistical link between the delay in decision to refer and intrapartum stillbirth.

Table 4 presents the association between the decision-making time to refer to YOTH and intrapartum stillbirth.

**Table 4:** Association between time to decision to refer and intrapartum fetal death.

Time between arrival at the health center and the decision to refer to the hospital	OR	95% IC		*p* value
Less than 1 h	3.11	1.39	6.94	0.005
1–2 h	2.83	1.07	7.46	0.035
>2 h	3.05	1.38	6.74	0.006

### 3.2. Time between the arrival of the patient at hospital YOTH and the first examination

There was no statistical association between the time to first examination at hospital and intrapartum stillbirth with *p* = 0.8.

### 3.3. Factors associated with intrapartum stillbirth in multivariate analysis

Table 5 presents the multivariate analysis of intrapartum stillbirth variables.

**Table 5:** Multivariate analysis of variables associated with intrapartum stillbirth.

Variables	OR (95% IC)	*p* value
Means of transportation to reach the hospital		
Ambulance/Emergency transportation	2.51 (0.55; 11.45)	0.236
Personal or private means		
Education level		
Some formal education		
No formal education	1.04 (0.73; 1.49)	0.829
Occupation		
Homemaker		
Civil servant/shopkeeper/student	1.07 (0.82; 1.53)	0.915
Time between the decision to seek care and arrival at the heath center CSPS		
Came directly to hospital		
Less than an hour	4.58 (2.55; 8.6)	1.03
1–2 h	5.02 (3.83; 10.45)	2.45
>2 h	4.48 (1.25; 11.08)	2.57
Time between arrival at the health center and the decision to refer to the hospital		
Less than an hour		
1–2 h	3.27 (2.09; 7.4)	1.59
>2 h	4.24 (1.11; 8.02)	2.05
Received an obstetric ultrasound during pregnancy		
No		
Second trimester	7.49 (6.40; 8.76)	<0.001
Third trimester	2.05 (0.51; 8.32)	0.315
Birth weight		
≥2,500 g		
<2,500 g	3.16 (2.11; 4.73)	<0.001
Person who mainly decided to seek care		
Patient		
Husband	2.94 (1.89; 4.56)	<0.001
In-laws/Family members	3.24 (2.29; 4.60)	<0.001
Number of pregnancies		
1		
3–4	1.27 (1.00; 1.62)	0.053
4 or more	1.96 (0.31; 12.28)	0.471
Previous caesarean section		
No	1.81 (1.77; 1.8)	<0.001
Yes		
Qualification of the agent who carried out the antenatal care		
Midwife		
Nurse	1.39 (1.12; 3.28)	0.843
Partogram		
No	1.67 (1.04; 3.56)	0.945
Yes		

## 4. Discussion

### 4.1. Limits and constraints of the study

The major difficulty of our study was the collection of information by interview with patients in emotional shock at the loss of the newborn.

### 4.2. Intrapartum stillbirth rate

The hospital intrapartum stillbirth rate was 20.1 per 1,000 births. This result is close to that of Sandjong et al. (12) in 2,009 in Cameroon who had found a rate of 18 per 1,000 births. On the other hand, it is clearly lower than that of Chigbu et al. (13) in 2009 in Nigeria who reported an intrapartum stillbirth rate of 52.1 per 1,000 births. In the United States of America, Fretts et al. (14) found an intrapartum stillbirth rate of 6.4‰ in 2005. In India, the intrapartum stillbirth rate was 16 per 1,000 births in 2014 (15). In view of these results, there is a disparity in intrapartum stillbirth rates from one country to another. The organization of care and the quality of the technical platform are explanatory elements.

### 4.3. Factors associated with socio-demographic characteristics

Patients under the age of 20 accounted for 19.3% (16) in the case group, a result similar to Foumane et al.'s study in Cameroon (16) and Getahun et al.'s study in Missouri (17) who found 18.9% and 19.5% of patients aged under 20, respectively, were victims of stillbirth. These findings suggest that young maternal age is a risk factor for intrapartum mortality. In fact, lack of reproductive experience is known to be associated with poor pregnancy outcomes (18, 19). Adolescents have been associated with adverse pregnancy outcomes with a decreasing risk of intrapartum stillbirth as maternal age decreases (19).

We found a predominance of intrapartum stillbirth in unmarried women similar to Foumane and colleagues (16). The intolerance of pregnancies outside of marriage in traditional African society causes these women to have hidden, unmonitored, high-risk pregnancies, and explains the maternal and fetal tragedy linked to these pregnancies. Nonetheless, marital status was not statistically related to the occurrence of intrapartum stillbirth. Wang (20) in his study also found no statistical link between marital status and stillbirth.

There was a statistically significant association between intrapartum stillbirth and patients with no financial income and no schooling. Lack of regular income and low level of education were identified as risk factors for intrapartum stillbirth by Foumane et al. in Cameroon. The poor are most at risk and have the lowest coverage of skilled birth care (1). Improving the socio-economic and educational status of women in developing countries could be a strategy to reduce the intrapartum stillbirth rate.

On univariate and multivariate analysis, there is a statistical link between intrapartum stillbirth and the decision to consult was either made by the spouse or the in-laws. Indeed, in a traditional African society woman have limited agency and decision-making power, especially within family dynamics. This can lead to a delay in the consultation.

### 4.4. Factors associated with clinical data

There was a statistical association (univariate analysis) between being referred and intrapartum stillbirth with a *p* = 0.016 with an OR of 0.51 (0.30; 0.88): patients that were referred for care at hospital were 3 times as likely to have intrapartum stillbirth. In multivariate analysis no statistical link was found (*p* = 0.236). This result corroborates that of Sandjong et al. in Cameroon (8). where the risk of stillbirth was high when the patient is referred by another health facility; [OR = 4.81, 95% CI (2.39–9.91); *p* < 0.001]. In Burkina Faso the problem lies in the inadequate evacuation conditions. Patients are referred when the conditions of the mother or the fetus are critical. Even more serious is the fact that the shortage of certain consumables in free care contributes to delaying the treatment of patients.

Antenatal consultations (ANC) were mainly carried out by midwives or gynecologists. The odds of intrapartum stillbirth was statistically significant when ANC consultations were performed by other health workers with *p* = 0.005 OR of 4.86 with a CI (1.63–14.50): patients who had ANC consultations were performed by other agents were four times as likely to have an intrapartum stillbirth. ANC consultations should be carried out by agents trained to meet the objectives. The unavailability of midwives at all times in peripheral health facilities could explain why ANC consultations are carried out by other agents (nurses, itinerant health agents).

There was a statistically significant association between patient's lack of obstetric ultrasound during pregnancy and intrapartum stillbirth (OR: 7.49; CI: 6.40, 8.76). Obstetric ultrasounds are a key intervention which can detect pregnancy complications and determining the prognosis for childbirth. Sandjong et al. have shown that a single ultrasound performed between the 28th and 32nd week of gestation would reduce intrapartum stillbirth (12).

### 4.5. Factors associated with delays

Bivariate analyses determined a statistical association between the delay in the decision to seek care at a health center and intrapartum stillbirth. Similarly, there was a statistical association between intrapartum stillbirth and delayed referral to hospital (greater than 2 h). On the other hand, we did not find a statistical association but this link disappears in the multivariate analysis. Chigbu et al.'s study in Nigeria found that all delays were associated with intrapartum stillbirth in both univariate and multivariate analysis (13).

## 5. Conclusion

The intrapartum stillbirth rate in our study remains high. Factors such as referral from a health facility, no obstetric ultrasound during pregnancy, low neonatal birth weight, and delay in decision-making to seek care were significantly associated with intrapartum stillbirth. Regular and effective follow-up of all pregnant women is essential through quality ANC and active labor and delivery monitoring using a partograph. Specific actions are necessary to enable rapid recognition of the risk factors for intrapartum stillbirth and to ensure rapid and effective management.

## Data Availability

The original contributions presented in the study are included in the article/Supplementary Material, further inquiries can be directed to the corresponding author.
